# Persistent racial and socioeconomic inequities in mycosis fungoides survival: a population-based study

**DOI:** 10.3389/fpubh.2026.1811186

**Published:** 2026-07-15

**Authors:** Tiantian Zhang, Simo Du, Weili Xue, Zhe Wang, Omer A. Idris, Cristina R. Bunton-Young

**Affiliations:** 1 Department of Internal Medicine, University of Central Florida/HCA, Pensacola, FL, United States; 2 Jacobi Medical Center - Albert Einstein College of Medicine, Bronx, NY, United States; 3 Department of Oncology, The First Affiliated Hospital of Zhengzhou University, Zhengzhou, China; 4 High Throughput Screening Core, Department of Share Resources, Beckman Research Institute of City of Hope, Duarte, CA, United States; 5 Department of Biological Science, Western Michigan University, Kalamazoo, MI, United States

**Keywords:** cutaneous T-cell lymphoma, machine learning, mycosis fungoides, prognostic modeling, racial disparities, socioeconomic status

## Abstract

Racial disparities in mycosis fungoides (MF) have been described historically, but their persistence in the contemporary era of diagnosis and care remains incompletely characterized. To address this gap, we evaluated survival disparities by race and socioeconomic status (SES) and assessed temporal trends in outcomes. To this end, we used the Surveillance, Epidemiology, and End Results (SEER)-22 database (2000–2021) to identify 13,694 patients with MF. Overall survival (OS) was assessed with Kaplan–Meier analyses and multivariable Cox proportional hazards models. Building on these traditional analyses, we also developed registry-based prognostic models using routinely collected variables with internal validation and geographic/registry-based external validation in a non-overlapping SEER registry subset. Across the study population, the overall 1-, 3-, and 5-year OS rates were 96.8%, 90.8%, and 85.8%, respectively. Survival was lower among Black and American Indian/Alaska Native (AI/AN) patients and highest among Asian/Pacific Islander (API) patients (5-year OS: 85.3% White, 82.7% Black, 92.9% API, 82.1% AI/AN). In multivariable adjusted analyses, Black race remained independently associated with higher mortality, whereas API race was associated with better survival. OS improved modestly in the post-2010 period overall, with a statistically significant improvement among White patients, while changes within other racial groups were not statistically significant. Importantly, lower SES independently predicted worse survival, and Black and AI/AN patients were disproportionately represented in lower-income quartiles; a similar SES distribution pattern was also observed in the National Inpatient Sample. In an integrative Cox model, Black or AI/AN race, older age, male sex, advanced stage, and lower SES independently predicted worse survival. Taken together, these findings indicate persistent racial and socioeconomic inequities in MF survival in the contemporary era. Interpretable prognostic models based on routinely collected variables may therefore support individualized risk stratification and inform efforts to improve equity in MF care.

## 1 Introduction

Mycosis fungoides (MF) is the most common subtype of cutaneous T-cell lymphoma (CTCL), accounting for more than half of all primary cutaneous lymphomas (1–3). In the U.S., CTCL occurs at a rate of roughly 7 new cases per million people each year, and patients are typically diagnosed at a median age of about 55–60 (4, 5). Although MF often follows an indolent course, outcomes are heterogeneous and vary by disease stage, access to care, and patient-level sociodemographic factors.

Multiple population-based studies have reported worse survival among Black patients compared with White patients, as well as differences in age at diagnosis and stage distribution, although findings have not been entirely consistent across datasets and eras (6–10). Socioeconomic status (SES) may contribute to these patterns through pathways such as healthcare access, delays in diagnosis, and differences in treatment availability; however, the extent to which SES explains or modifies racial/ethnic survival disparities in MF remains incompletely characterized (11). Moreover, whether disparities have changed over time in the contemporary era has not been systematically evaluated in a large, nationally representative cohort.

Over the past two decades, both the therapeutic and policy landscapes for MF have evolved considerably. The introduction of histone deacetylase (HDAC) inhibitors (vorinostat, 2006; romidepsin, 2009) and, later, targeted biologic therapies (brentuximab vedotin, 2017; mogamulizumab, 2018) has expanded systemic options for relapsed or refractory disease (12–15). In parallel, broader changes in healthcare delivery and coverage [including implementation of the Affordable Care Act (ACA) in 2010] and increasing standardization of clinical guidelines may have influenced population-level patterns of care (16). However, Black patients and other historically underserved groups have been underrepresented in many CTCL clinical studies, raising concerns about the generalizability of treatment trial evidence and the equity of real-world benefit (8).

Concurrently, prognostic modeling has been developed for a range of solid and hematologic malignancies, such as breast, bladder, and soft-tissue sarcomas, to support individualized risk stratification and treatment decisions (17–19). MF, despite marked clinical heterogeneity, lacks widely adopted and externally validated prognostic tools that rely on routinely collected variables. Prediction methods, including penalized regression and other machine-learning approaches, can complement conventional epidemiologic modeling by improving risk stratification in large observational datasets and by providing calibrated survival estimates when rigorously validated (20). However, their utility in MF and their generalizability across populations and settings remain uncertain.

In this study, we addressed both descriptive and implementation gaps in MF outcomes research. Using the Surveillance, Epidemiology, and End Results (SEER)-22 database (2000–2021), we characterized racial/ethnic and SES differences in patient profiles, quantified survival disparities, and evaluated temporal trends, including comparisons before and after 2010. We then assessed whether similar socioeconomic patterning was observed in an independent inpatient dataset [the 2022 National Inpatient Sample (NIS)]. Finally, as a secondary objective, we developed an interpretable prognostic model using routinely available registry variables, performed internal validation in a SEER-22 development cohort excluding SEER-8 registries, and conducted geographic/registry-based external validation in SEER-8. Together, these analyses aim to clarify persistent inequities in MF outcomes and to provide a practical risk stratification tool to support equity-focused efforts in MF care.

## 2 Methods and materials

### 2.1 Data source and cohort definition

Patients with mycosis fungoides were identified from the SEER Research Limited-Field Data, 22 Registries (excl IL and MA), Nov 2023 sub (2000–2021) using ICD-O-3 histology code 9700 and malignant behavior code 3 (ICD-O-3 9700/3). The primary cohort was restricted to patients diagnosed from January 1, 2000, through December 31, 2021. Cases identified only by death certificate or autopsy were excluded.

Because SEER contains tumor-level rather than one-record-per-patient data, we used the “Sequence number central” variable to include MF recorded as “one primary only” or “first of two or more primaries.” MF occurring as a second or subsequent reportable primary malignancy was excluded. The resulting cohort contained 13,694 patients for the principal demographic, survival, temporal, and socioeconomic analyses. Demographic variables included age, sex, race, year of diagnosis, and marital status. Race categories were White, Black, Asian/Pacific Islander (API), American Indian/Alaska Native (AI/AN), and Unknown. Race and Hispanic origin were not combined into mutually exclusive race/ethnicity categories in the primary analysis; therefore, the White category included patients classified as White regardless of Hispanic ethnicity.

For complementary SES profiling in an independent inpatient dataset, we queried the 2022 National Inpatient Sample (NIS) for hospitalizations with MF (ICD-10 C84.0x). NIS variables included race/ethnicity, age, income quartile (based on ZIP-code–level median income), and health insurance status, represented by the primary expected payer. Missingness was evaluated separately for race/ethnicity, ZIP-code income quartile, and health insurance/primary expected payer. Records missing a variable were excluded only from analyses involving that variable, and no values were imputed.

Disease extent was characterized using the SEER-provided Combined Summary Stage variable and categorized as localized, regional, distant, or unknown/unavailable. These categories broadly represent disease confined to the skin, more extensive cutaneous disease and/or lymph-node involvement, and visceral or other distant-organ involvement, respectively. Unrecorded values were retained as unknown/unavailable. We used the categories as provided by SEER and did not construct an investigator-defined stage crosswalk.

Socioeconomic context was represented by county-level median household income linked to the patient's county of residence. This measure was derived from the 2017–2021 American Community Survey, Table B19013, and expressed in 2021 inflation-adjusted U.S. dollars. Income was analyzed using the SEER-defined categories of < $50,000, $50,000–59,999, $60,000–69,999, $70,000–79,999, $80,000–94,999, and ≥$95,000, with the highest-income category serving as the reference group in regression analyses. County-level income was selected because it was consistently available across participating SEER registries and provided a standardized measure of residential socioeconomic context. It should be interpreted as an area-level contextual measure rather than as the income or socioeconomic circumstances of an individual patient. A usable county identifier was not available in the analytic dataset; therefore, standard errors could not be clustered at the county level.

For prognostic model development, patients from registries included in SEER-8 were excluded from the SEER-22 cohort to prevent registry overlap with the external-validation population, resulting modeling cohort included 10,650 patients, of whom 7,747 diagnosed through 2016 were assigned to the training cohort and 2,903 diagnosed after 2016 were assigned to the held-out temporal test cohort.

External registry-based validation was conducted using the SEER Research Data, 8 registries, Nov 2023 Sub (1975–2021). MF cases were identified using the same ICD-O-3 histology and behavior definitions, and eligibility criteria and predictor definitions were harmonized with the development cohort. A total of 4,753 patients were included in the external-validation cohort. The complete case-selection process is shown in Supplementary Figure 1, and the exact database titles, registry composition, variable definitions, and coding rules are provided in Supplementary Table 1.

### 2.2 Descriptive and survival analyses

Descriptive statistics summarized patient demographics and clinical characteristics. Continuous variables were compared using the Kruskal–Wallis test, and categorical variables with χ^2^ or Fisher's exact test. Overall survival (OS) was defined from diagnosis to death from any cause or last follow-up. Survival probabilities were estimated using the Kaplan–Meier method and compared across races using the log-rank test. Time-specific OS at 1, 3, and 5 years was calculated with Greenwood confidence intervals.

### 2.3 Cox regression modeling

Cox proportional-hazards models were used to estimate hazard ratios (HRs) and 95% confidence intervals (CIs) for mortality associated with race. We also fit a series of sequentially adjusted models: Model 0 was unadjusted; Model 1 adjusted for age, sex, and year of diagnosis; Model 2 added SES; Model 3 added disease stage; and Model 4 was a 1-year landmark analysis excluding patients who died within 12 months of diagnosis. Proportional hazards assumptions were verified using Schoenfeld residuals.

### 2.4 Temporal trend and socioeconomic analyses

To evaluate temporal trends, patients were stratified by diagnosis era (2000–2010 vs. 2011–2021). Kaplan–Meier curves and global log-rank tests compared OS between eras overall and within racial groups. As secondary analyses, 12-, 36-, and 60-month OS estimates were compared between eras within each racial group using Greenwood standard errors, with Holm adjustment applied across the 15 resulting comparisons.

To assess whether the association between diagnosis era and OS differed by race, we compared unadjusted Cox models with and without race-by-era interaction terms using a likelihood-ratio test. Patients with Unknown race were excluded because this category reflects missing racial classification. The 2010 cutoff was prespecified, and temporal findings were interpreted descriptively rather than attributed to specific treatments or policies.

For SES analyses, county-level income quartiles were compared across races in SEER, and Fisher's exact tests with simulated *p*-values assessed distributional differences. NIS results were summarized by race for age, income quartile, and payer type, using z-tests to compare proportions.

### 2.5 Prognostic modeling and validation

As a secondary objective, we developed prognostic models for OS using routinely collected registry variables. The SEER-22 development cohort included MF cases with complete data on age, sex, race/ethnicity, SEER Summary Stage, county-level income quartile (IncomeGroup), rural–urban residence (RuralCode), and diagnosis period ( ≤ 2010 vs. 2011+). To ensure non-overlap with the external validation cohort, all SEER-8 registries were excluded from the SEER-22 development cohort.

We fit three survival models: (1) penalized Cox regression with an L1 penalty (LASSO-Cox), (2) random survival forest (RSF), and (3) gradient-boosted Cox model (XGBoost-Cox). Vital status was harmonized to a binary event indicator after internal plausibility checks of event prevalence and short-term OS.

For internal validation, we used a temporal split by year of diagnosis, with cases diagnosed ≤ 2016 assigned to the training set and those diagnosed after 2016 forming a held-out test set. Hyperparameters for LASSO-Cox, RSF, and XGBoost-Cox were tuned using K-fold cross-validation within the training data.

Model discrimination in the SEER-22 test set was evaluated using Harrell's C-index and Uno's time-dependent area under the ROC curve (AUC) at prespecified horizons (12, 24, 36, and 48 months). Prediction error was assessed using Brier scores at the same time points. Calibration for the LASSO-Cox model was examined by comparing observed versus predicted risk across deciles of predicted risk; time-specific Brier scores from the Score framework provided complementary calibration assessment across models.

External validation was performed in a non-overlapping SEER-8 (1975-2022) cohort restricted to MF (ICD-O-3 9700/3) with harmonized definitions of age, sex, race/ethnicity, Summary Stage, IncomeGroup, RuralCode, and diagnosis period. Models trained in SEER-22 were applied to SEER-8 without refitting. External performance was quantified using Harrell's C-index, Uno time-dependent AUC, and Brier scores at 12, 24, 36, 48, and 60 months. The purpose of external validation was not to reconfirm that individual variables such as age and stage are associated with survival. Rather, it was to determine whether the complete locked prediction function—including the estimated coefficients, baseline hazard, risk ranking, and absolute survival estimates—retained discrimination and calibration when applied without refitting to non-overlapping registry populations.

### 2.6 Web-based risk calculator

To facilitate individualized prognostication, we implemented an interactive web-based calculator in R Shiny based on the final locked LASSO-Cox model. The application accepts the same variables available at diagnosis (age, sex, race/ethnicity, Summary Stage, income quartile, rural–urban residence, and diagnosis period) and returns predicted OS probabilities at 12, 24, 36, 48, and 60 months. Predictions are generated by mapping categorical levels to those used during model training, calculating the linear predictor from locked coefficients, and converting this to absolute survival probabilities using the baseline cumulative hazard estimated in the training cohort. The calculator is available at: https://tzhang902.shinyapps.io/mf_calculator/. The calculator is intended for research and exploratory risk-stratification purposes. Its estimates are based on registry-level variables and should not be used as a substitute for formal clinical staging, treatment-specific assessment, specialist evaluation, or individualized clinical judgment.

### 2.7 Software and statistical analyses

All analyses were performed using R version 4.3.0 (R Foundation for Statistical Computing, Vienna, Austria). Statistical significance was set at *p* < 0.05. All tests were two-sided, and statistical significance was defined as *p* < 0.05. Holm's method was applied only to the 15 secondary race-specific pointwise comparisons of 12-, 36-, and 60-month OS between diagnosis eras. Global log-rank tests, Cox regression analyses, and the prespecified global race-by-era interaction test were not included in this multiple-testing adjustment.

### 2.8 Ethical approval

The Institutional Review Board (IRB) of University of Central Florida/HCA Healthcare determined that this research is exempt or excluded from IRB oversight as the study posed minimal risk and utilized securely de-identified data. SEER data were accessed under a research data agreement beginning 01/01/2025. The investigators did not access identifiable participant information at any time.

## 3 Results

### 3.1 Cohort composition and demographic patterns

We first characterized the demographic and clinical composition of patients with MF in the SEER-22 database to establish the population framework for subsequent analyses. A total of 13,694 patients diagnosed between 2000 and 2021 were identified. The median age at diagnosis was 57 years (range, 1–90 years) and differed significantly by race (*p* < 0.0001). Median age was highest among White (59 years) and AI/AN (57 years) patients, and lowest among API (48 years) and Unknown race (49 years) groups, followed by Black patients (52 years).

Across racial groups, the proportion of patients diagnosed before age 60 differed markedly (*p* < 0.0001). Black and API patients were most likely to be diagnosed at a younger age (< 60 years: 69.7% and 72.0%, respectively), compared with White patients (50.5%).

Sex distribution also varied significantly by race (*p* < 0.0001). MF showed an overall male predominance, but this pattern differed by group: White (58.2% male), API (55.7%), and AI/AN (51.8%) patients were predominantly male, whereas Black patients showed a slight female predominance (53.7% female) (Table 1).

**Table 1:** Demographic summary of mycosis fungoides by race.

Race	*N*	Median age (yrs)	Age range (yrs)	% < 60 yrs	% ≥60 yrs	% Male	% Female
All race	13,694	57	1–90	56.2	43.8	55.8	44.2
White	9 667	59	1–90	50.5	49.5	58.2	41.8
Black	2 393	52	2–90	69.7	30.3	46.3	53.7
Asian/Pacific Islander	853	48	1–90	72.0	28.0	55.7	44.3
American Indian/Alaska Native	56	57	8–84	58.9	41.1	51.8	48.2
Unknown	725	49	6–90	69.1	30.9	55.6	44.4

### 3.2 Survival outcomes by race

We next examined survival outcomes to assess whether demographic differences translated into survival disparities among racial groups. At a median follow-up of 104 months, overall 1-, 3-, and 5-year survival rates for MF patients with all races were 96.8%, 90.8%, and 85.8%, respectively (Table 2A).

**Table 2A:** Overall and race-specific overall survival, SEER-22, 2000–2021.

Race	12-mo OS (%) [95% CI]	36-mo OS (%) [95% CI]	60-mo OS (%) [95% CI]	Median OS (mo)
All races	96.8 (96.5–97.1)	90.8 (90.3–91.4)	85.8 (85.1–86.4)	NR
White	96.8 (96.5–97.2)	90.5 (89.9–91.1)	85.3 (84.6–86.1)	NR
Black	95.5 (94.6–96.3)	89.0 (87.7–90.3)	82.7 (81.1–84.4)	NR
American Indian/Alaska Native	94.5 (88.7–100)	88.6 (80.5–97.6)	82.1 (72.1–93.5)	154.0 (119.0-NA)
Asian/Pacific Islander	98.1 (97.1–99.0)	95.3 (93.8–96.8)	92.9 (91.0–94.9)	NR
Unknown	99.3 (98.7–100)	98.0 (96.8-99.2)	96.3 (94.6–98.1)	NR

The first row (“Overall”) summarizes total SEER22 MF cohort; subsequent rows show race-stratified OS and median OS.

**Figure 1:**
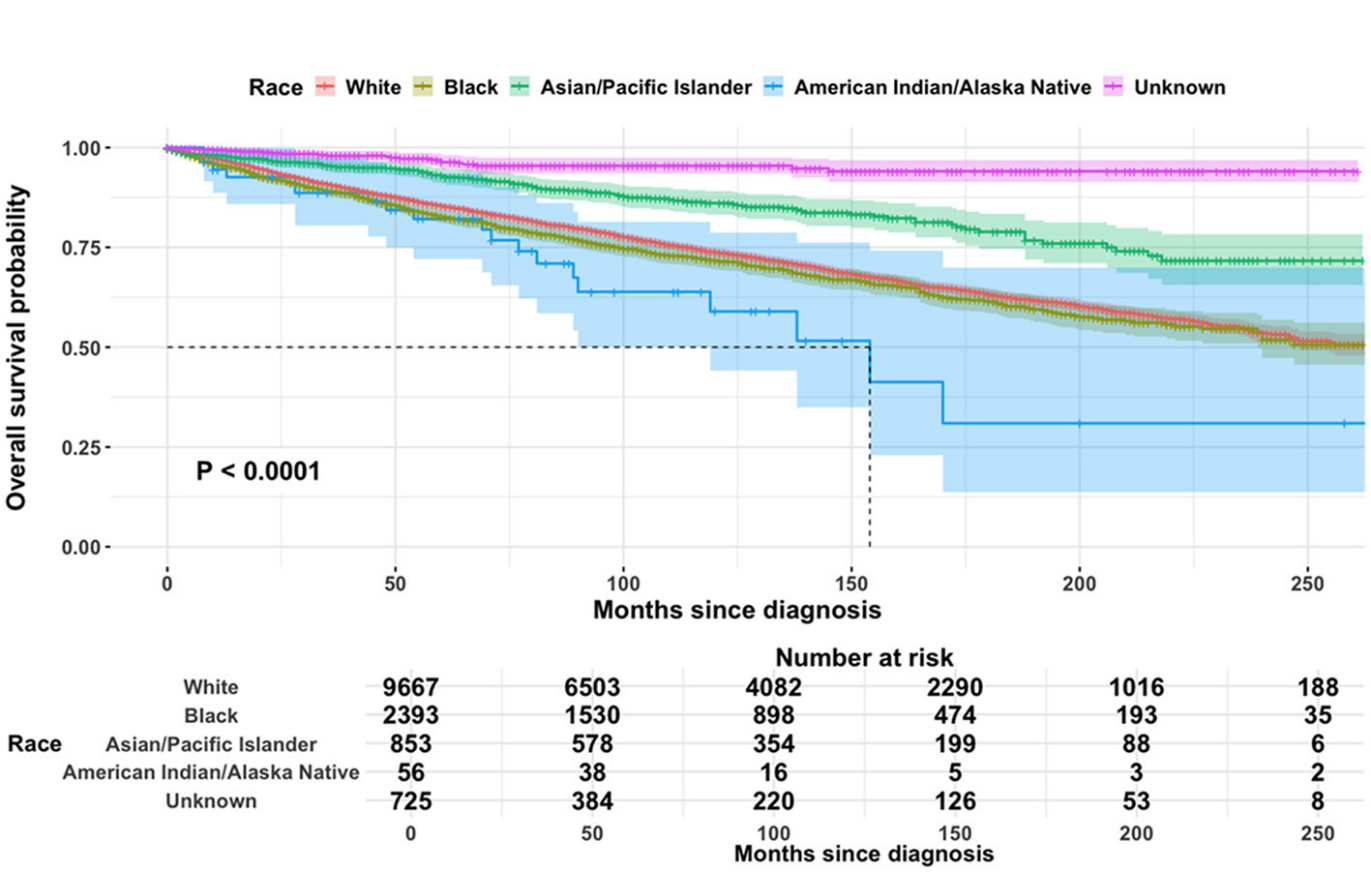
Overall survival of mycosis fungoides by race. Kaplan–Meier curves display overall survival among patients with MF, stratified by race (White, Black, API, American Indian/Alaska Native, and Unknown). Each step downward represents an event (death), while vertical tick marks indicate censored observations (patients alive or lost to follow-up at that time). Shaded regions denote 95% confidence intervals. The number of patients at risk is shown below the x-axis at 24-month intervals. Median survival is indicated by horizontal and vertical dashed lines. A global log-rank test demonstrated a statistically significant difference in survival among racial groups (*p* < 0.0001), with Black patients exhibiting the lowest overall survival and API patients showing the most favorable outcomes over time. Estimates for the Unknown race category should be interpreted cautiously because this category represents missing classification rather than a defined racial population.

When stratified by race, survival remained high across all groups but was consistently lower among Black patients compared with White patients and most favorable among API patients. At 36 months, OS was 89.0% for Black patients vs. 90.5% for White patients; at 60 months, 82.7% for Black vs. 85.3% for White. In contrast, API patients had the highest survival at each time point (98.1%, 95.3%, and 92.9% at 12, 36, and 60 months, respectively). AI/AN patients showed the lowest long-term point estimates among named race groups (e.g., 82.1% at 60 months; median OS 154.0 months [95% CI, 119.0–NA]), though precision was likely limited by small numbers (Table 2A; Figure 1).

Cox regression confirmed that Black race was independently associated with significantly higher mortality compared with White race, with unadjusted hazard ratios of 1.45 (95% CI 1.16–1.82) at 1 year and 1.20 (1.07–1.36) at 5 years. After adjustment for age, sex and year, the disparity persisted and became more pronounced, with adjusted HRs of 2.52 (2.01–3.17), 1.98 (1.71–2.29), and 2.00 (1.77–2.26) at 1, 3, and 5 years, respectively. Conversely, API patients retained a modest survival advantage after multivariable adjustment [HR 0.74 (0.56–0.98) at 5 years]. AI/AN patients trended toward worse survival than White patients across models, but estimates did not reach statistical significance [e.g., unadjusted 60-month HR 1.26 (0.65–2.43); adjusted 1.43 (0.74–2.75)] (Table 2B).

**Table 2B:** hazard ratios for overall survival by race (vs. White), SEER-22, 2000–2021.

Race	12-mo HR (95% CI)	36-mo HR (95% CI)	60-mo HR (95% CI)	Adjusted 12-mo HR (95% CI)	Adjusted 36-mo HR (95% CI)	Adjusted 60-mo HR (95% CI)
Black	1.45 (1.16–1.82)	1.18 (1.03–1.37)	1.20 (1.07–1.36)	2.52 (2.01–3.17)	1.98 (1.71–2.29)	2.00 (1.77–2.26)
American Indian/Alaska Native	1.75 (0.56–5.44)	1.23 (0.55–2.76)	1.26 (0.65–2.43)	2.03 (0.65–6.33)	1.41 (0.63–3.14)	1.43 (0.74–2.75)
Asian/Pacific Islander	0.63 (0.38–1.04)	0.50 (0.36–0.69)	0.47 (0.35–0.62)	1.05 (0.64–1.74)	0.81 (0.58–1.13)	0.74 (0.56–0.98)
Unknown	0.21 (0.08–0.55)	0.21 (0.11–0.38)	0.22 (0.14–0.36)	0.38 (0.14–1.02)	0.36 (0.20–0.64)	0.38 (0.23–0.61)

Interpretation: HR > 1 indicates higher mortality compared with White patients. Adjusted models include age, sex and year.

Sequential multivariable modeling further clarified the durability and magnitude of racial disparities in overall survival (Table 2C). In the crude model, Black patients overall had a 1.11-fold higher hazard of death compared with White patients (95% CI, 1.01–1.21). Adjustment for age, sex, and year strengthened this association (HR 1.87, 95% CI 1.70–2.05), and the excess mortality persisted after additional adjustment for SES [HR 1.86, 95% CI 1.69–2.05] and disease stage (HR 1.79, 95% CI 1.63–1.97). Even after excluding early deaths using a 1-year landmark analysis, the disparity remained significant (HR 1.76, 95% CI 1.60–1.94). API patients consistently exhibited more favorable outcomes, with hazards ranging from 0.51 (0.42–0.62) in the crude model to 0.75 (0.61–0.92) after full adjustment, indicating a persistent survival advantage. AI/AN patients showed a trend toward poorer survival than White patients, with hazard ratios between 1.52 (0.94–2.46) and 1.74 (1.11–2.74) across models, though estimates did not reach statistical significance due to small sample size.

**Table 2C:** Sequential multivariable cox models for overall survival by race, SEER-22, 2000–2021.

Term	Model 0: crude	Model 1: + age, sex, year	Model 2: + SES	Model 3: + stage	Model 4: + 1Y landmark
Black	1.11 (1.01–1.21)	1.87 (1.70–2.05)	1.86 (1.69–2.05)	1.79 (1.63–1.97)	1.76 (1.60–1.94)
American Indian/Alaska Native	1.63 (1.04–2.56)	1.74 (1.11–2.74)	1.52 (0.94–2.46)	1.52 (0.94–2.46)	1.58 (0.98–2.56)
Asian/Pacific Islander	0.51 (0.42–0.62)	0.76 (0.63–0.93)	0.81 (0.67–0.99)	0.80 (0.65–0.97)	0.75 (0.61–0.92)
Unknown	0.16 (0.10–0.24)	0.26 (0.17–0.40)	0.27 (0.18–0.41)	0.28 (0.19–0.43)	0.27 (0.18–0.42)

Sequential Cox proportional-hazards models evaluating overall survival by race. Model 0 = unadjusted; Model 1 = adjusted for age, sex, and year of diagnosis; Model 2 = additionally adjusted for SES; Model 3 = further adjusted for disease stage; Model 4 = 1-year landmark analysis excluding early deaths. HRs are shown with 95% confidence intervals in parentheses.

Taken together, these results confirm that racial survival disparities in MF remain substantial and persistent across all analytic frameworks. Adjustment for SES (Table 2C, Model 2) and clinical stage (Table 2C, Model 3) modestly attenuated but did not eliminate the excess mortality among minority patients, suggesting that socioeconomic disadvantage is an important but incomplete mediator. Meanwhile, the consistently favorable outcomes among API patients and the trend toward poorer outcomes among Black and AI/AN patients highlight the complex interplay of biologic, structural, and healthcare-access factors that continue to shape inequities in MF outcomes.

### 3.3 Temporal trends in survival and the modern treatment era

**Figure 2:**
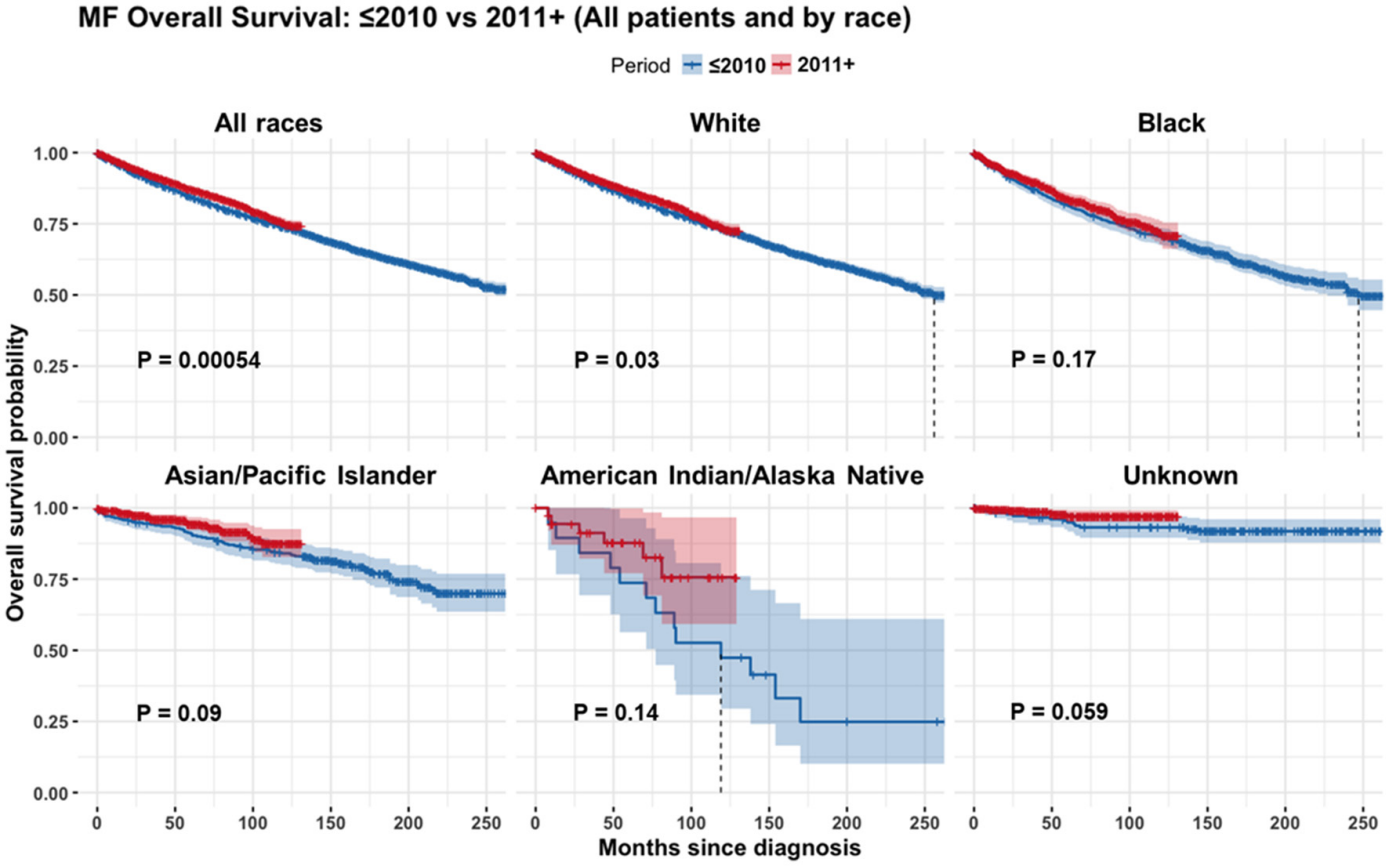
Overall survival of patients with mycosis fungoides diagnosed before and after 2010, overall and by race. Kaplan–Meier curves depict overall survival for patients diagnosed in 2000–2010 (blue) and 2011–2021 (red) in the SEER-22 database. The upper-left panel shows the overall cohort, and the remaining panels display results stratified by race. A significant improvement in survival after 2010 was observed in the overall population (*p* < 0.0001) and among White patients (*p* = 0.03), while survival gains for Asian/Pacific Islander (*p* = 0.14), Black (*p* = 0.17), and American Indian/Alaska Native (*p* = 0.059) patients were not statistically significant. The Unknown race group showed no notable difference between eras. Shaded areas represent 95% confidence intervals.

To evaluate temporal changes in survival before and after 2010, a period corresponding to the adoption of HDAC inhibitors and later biologic therapies, we compared survival before and after 2010, the period marking widespread adoption of HDAC inhibitors and biologic therapies (Table 3, Figure 2). Across the entire cohort, survival improved modestly in the modern treatment era. Five-year OS increased from 84.4% to 87.1% (+2.7 percentage points), representing a statistically significant improvement (p < 0.001). One- and three-year OS also rose slightly from 96.3% to 97.1% and 89.8% to 91.7%, respectively, with all three differences reaching statistical significance (Supplementary Table 2). The Kaplan–Meier curves demonstrate a consistent upward shift in survival for the 2011+ cohort, confirming a measurable improvement across time (Figure 2).

**Table 3:** Overall and race-specific 12-, 36-, and 60-month overall survival before and after 2010.

Race/Period	12-mo OS (%) [95% CI]	36-mo OS (%) [95% CI]	60-mo OS (%) [95% CI]
Overall ( ≤ 2010)	96.3 (95.8–96.8)	89.8 (89.0–90.6)	84.4 (83.4–85.3)
Overall (2011+)	97.1 (96.7–97.5)	91.7 (91.1–92.4)	87.1 (86.2–87.9)
White ( ≤ 2010)	96.5 (96.0–97.1)	89.7 (88.8–90.6)	84.1 (83.0–85.3)
White (2011+)	97.1 (96.7–97.4)	91.2 (90.4–92.0)	86.5 (85.5–87.6)
Black ( ≤ 2010)	94.9% (93.5–96.4)	87.6% (85.5–89.8)	81.4% (78.9–83.9)
Black (2011+)	95.8% (94.7–96.9)	90.0% (88.4–91.7)	83.6% (81.4–85.9)
Asian/Pacific Islander ( ≤ 2010)	96.5% (94.4–98.5)	94.2% (91.6–96.8)	90.9% (87.8–94.2)
Asian/Pacific Islander (2011+)	99.0% (98.2–99.9)	95.8% (94.0–97.8)	94.4% (92.1–96.8)
American Indian/Alaska Native ( ≤ 2010)	94.7% (85.2–100.0)	84.2% (69.3–100.0)	73.7% (56.3–96.4)
American Indian/Alaska Native (2011+)	94.4% (87.1–100.0)	91.2% (82.2–100.0)	87.7% (77.0–99.8)
Unknown ( ≤ 2010)	98.9% (97.3–100.0)	96.6% (94.0–99.3)	94.9% (91.7–98.2)
Unknown (2011+)	99.5% (98.9–100.0)	98.6% (97.4–99.8)	97.1% (95.1–99.2)

OS, Overall Survival. Data are shown for all races pooled (top rows) and by race before ( ≤ 2010) and after (2011+) 2010. 95% confidence intervals are provided in parentheses.

When stratified by race, the magnitude and significance of improvement varied. White patients showed significant gains at both 3 and 5 years (89.7% → 91.2%, *p* = 0.017; 84.1% → 86.5%, *p* = 0.002), while the 1-year increase (96.5% → 97.1%) was not statistically significant. Black patients demonstrated a smaller, non-significant improvement (81.4% → 83.6% at 5 years, *p* = 0.19). The survival curves for API (*p* = 0.09) and AI/AN (*p* = 0.14) groups also trended upward but did not reach statistical significance, likely reflecting smaller sample sizes and wider confidence intervals. The Unknown category remained unchanged between periods (Supplementary Table 2).

The global race-by-diagnosis-era interaction was not statistically significant (likelihood-ratio χ^2^ = 4.83, 3 degrees of freedom, *p* = 0.185), indicating no evidence that the association between diagnosis era and OS differed by race. In era-specific adjusted contrasts, Black patients had significantly higher mortality than White patients in both 2000–2010 (HR, 1.77; 95% CI, 1.57–1.99; *p* < 0.001) and 2011–2021 (HR, 1.69; 95% CI, 1.46–1.96; *p* < 0.001). The Black race-by-era interaction was not significant (interaction HR, 0.96; 95% CI, 0.79–1.15; *p* = 0.643), indicating that the magnitude of the Black–White mortality disparity did not significantly change between eras (Supplementary Table 3). Thus, survival improved after 2010, but the adjusted mortality disadvantage among Black patients persisted, underscoring the need to further investigate socioeconomic and structural factors that may underlie the persistent survival gap.

### 3.4 Socioeconomic inequality and racial disparities in SEER

To investigate potential contributors to racial disparities, we next examined the distribution of SES across racial groups within SEER-22. Income distributions differed significantly across racial groups (global Fisher's exact test with simulation, *p* = 2 × 10?4). Black and AI/AN patients were over-represented in lower-income strata, with 11.2% and 7.1% respectively in the < $50k category, compared with 4.8% among White and 1.1% among API patients. At the upper end, API patients had the largest share in the ≥$95k category (45.4%), followed by White (28.0%), whereas Black (17.6%) and AI/AN (14.3%) patients were least represented (Supplementary Table 4). These patterns indicate meaningful SES heterogeneity by race and support evaluating socioeconomic factors alongside race in survival models; notably, racial survival differences persisted even when models adjusted for SES (Table 2C), underscoring that socioeconomic disadvantage is a contributor but not a complete explanation for outcome gaps.

### 3.5 Validation of SES using NIS data

**Figure 3:**
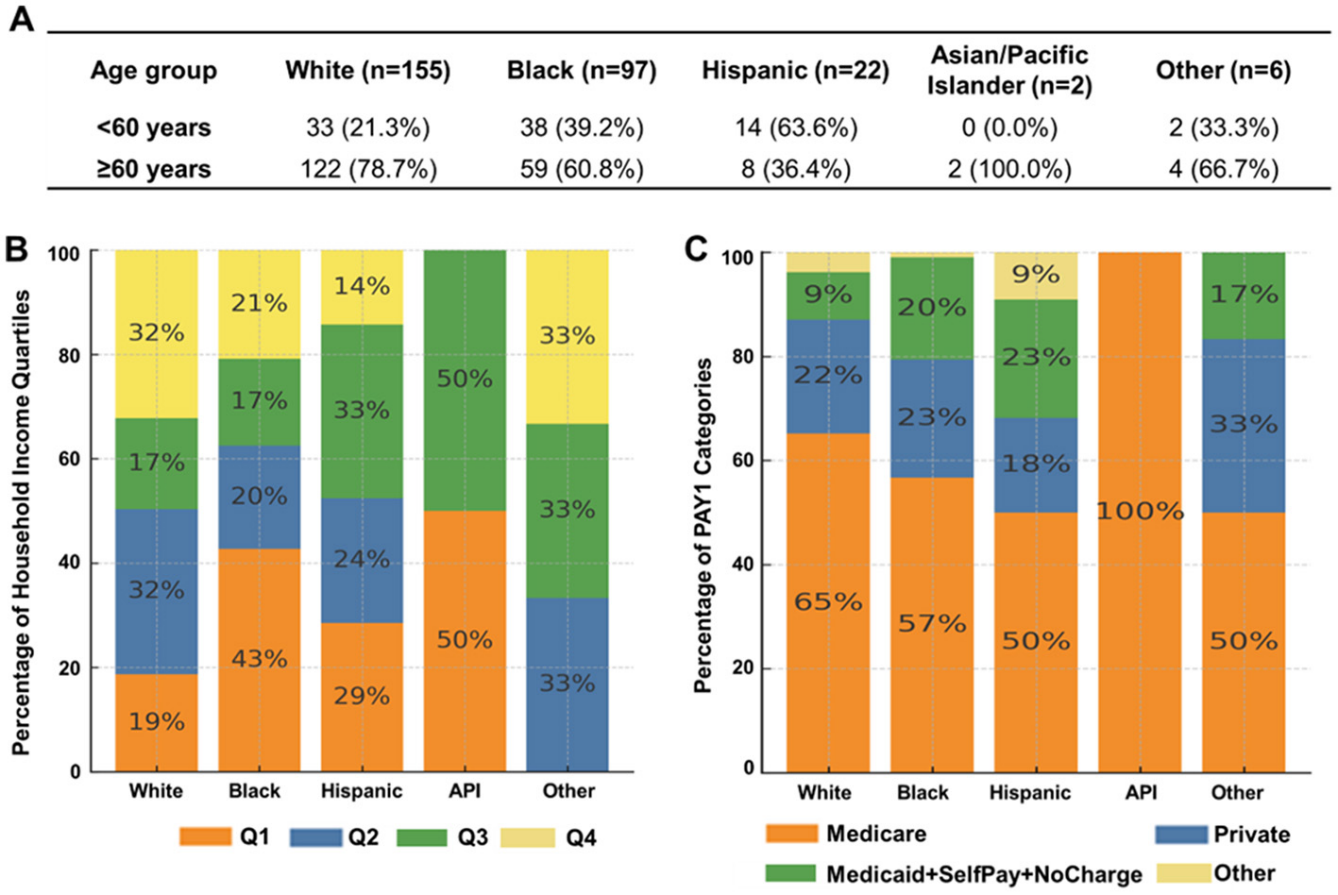
Socioeconomic and demographic distribution of Mycosis Fungoides hospital discharges in the NIS, 2022. **(A)** Age distribution by race, showing the proportion of patients aged <60 and ≥60 years. **(B)** Household income quartile by race based on the median income of the patient's ZIP code (Q1 = lowest, Q4 = highest). **(C)** Payer mix by race, combining Medicare (orange), Private (blue), Medicaid + Self-pay + No-charge (green), and Other (yellow) coverage types, illustrating greater reliance on public or uninsured payer sources among Black patients. All bars represent 100% stacked proportions within each racial group.

To validate socioeconomic differences in an independent dataset, we analyzed hospitalized MF discharges from the 2022 NIS. As shown in Figure 3A, age distribution varied by race. Black patients with MF were substantially younger than White patients, with 39% Black patients and 64% Hispanic patients < 60 years old, compared with only 21% among White patients, which is consistent with prior observations of earlier disease onset in minority groups.

As shown in Figure 3B, Black patients were disproportionately represented in the lowest income quartile (Q1) compared with White patients (43% vs. 19%, *p* < 0.001), indicating significantly higher residence in low-income ZIP codes. No statistically significant difference was observed between Black and Hispanic patients (43% vs. 29%, *p* = 0.19), nor between Black and API (50.0%, *p* = 0.83). When the lower two income quartiles (Q1 + Q2) were combined, the proportion remained higher among Black patients compared with White patients (63% vs. 51%), although the difference did not reach statistical significance (*p* = 0.074). These reflect persistently lower household income levels among Black patients.

Consistent with income distribution, payer mix also reflected socioeconomic disparities (Figure 3C). Among MF discharges, Black patients had a significantly higher proportion of Medicaid, self-pay, or no-charge coverage compared with White patients (20% vs. 9%, *p* = 0.026), supporting greater economic disadvantage. By contrast, the difference between Black and Hispanic patients (20% vs. 23%) was not statistically significant (*p* = 0.97). Across all races, Medicare predominated among older populations, while private insurance was more common among middle-income and working-age patients. By contrast, Medicaid and self-pay status served as strong indicators of socioeconomic vulnerability, reinforcing the relationship between payer type and income-based health inequities.

Taken together, these findings from the NIS dataset independently validate that Black patients with MF are more likely to be younger, reside in lower-income areas, and rely on public or non-private insurance coverage, reinforcing the role of socioeconomic disadvantage as a structural determinant of unequal outcomes.

### 3.6 Integrated predictors of survival

**Figure 4:**
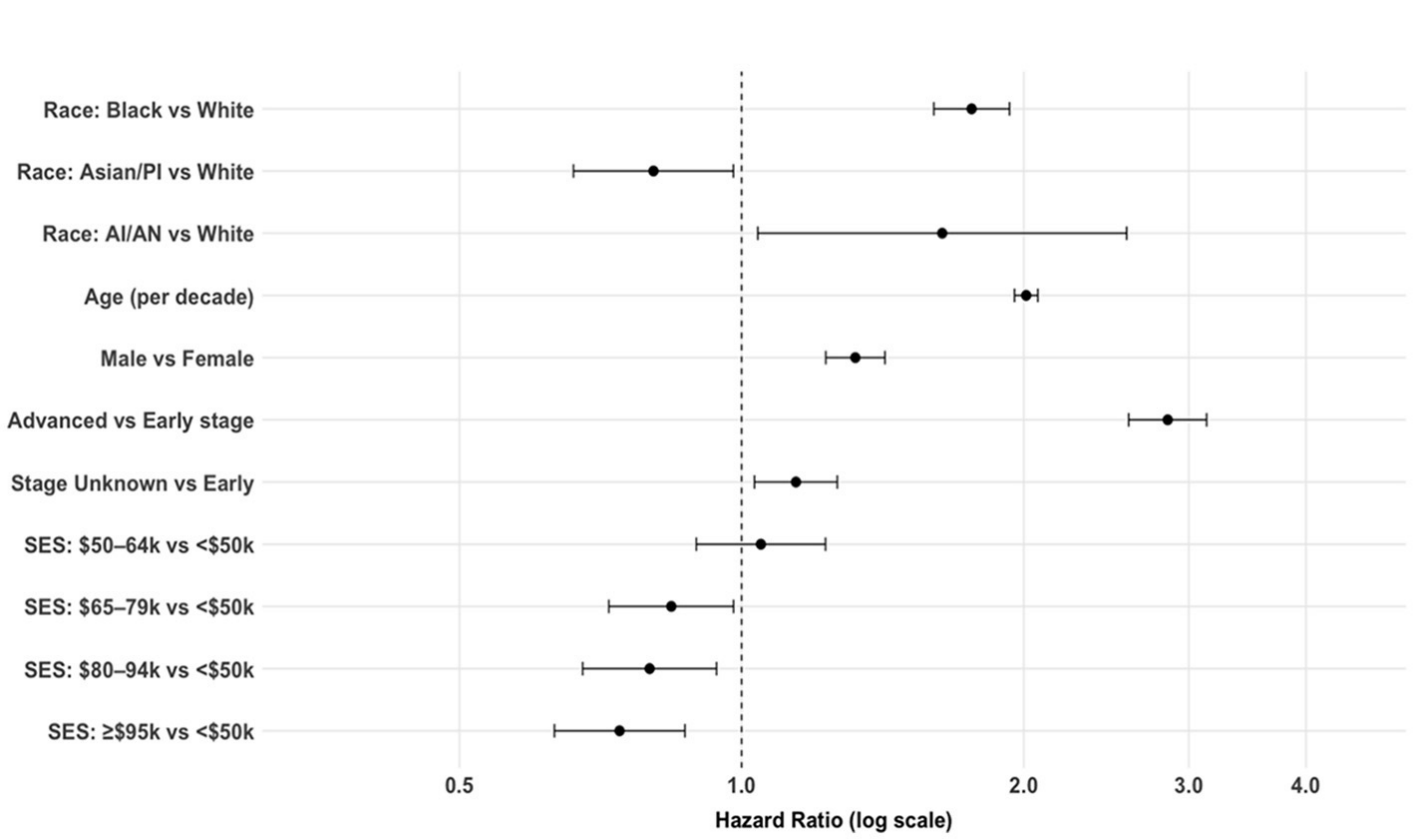
Independent predictors of overall survival in patients with mycosis fungoides (multivariable Cox model). Forest plot depicting HRs with 95% confidence intervals for key demographic, clinical, and socioeconomic predictors of overall survival. Models were adjusted for age, sex, stage, race, and county-level income quartile. Error bars represent 95% confidence intervals on a log scale.

To quantify the independent contributions of demographic, clinical, and socioeconomic factors, we fit a fully adjusted multivariable Cox proportional hazards model including race/ethnicity, age, sex, disease stage, and county-level income quartile (Figure 4).

In integrative model, Black race remained independently associated with significantly higher mortality compared with White race (adjusted HR = 1.76, 95% CI 1.60–1.93), while API patients continued to show a survival advantage (adjusted HR = 0.81, 95% CI 0.66–0.98). Older age and advanced-stage disease were strong predictors of death, and male sex was modestly but significantly associated with poorer survival.

A clear socioeconomic gradient was also observed: patients from lower-income counties (< $50k) had significantly higher hazards of death compared with those from the highest-income quartile (≥$95k), and mortality decreased progressively across higher income levels. Collectively, race, disease stage, sex, age, and area-level SES were independently associated with survival in the multivariable model. These findings suggest that observed MF survival disparities are associated with both clinical characteristics and sociodemographic context; however, the observational design and absence of detailed treatment, comorbidity, and healthcare-access data preclude causal interpretation.

### 3.7 Development of machine learning-based survival prediction model

For prognostic modeling, we used the SEER-22 MF cohort after excluding SEER-8 registries to ensure non-overlapping development and validation populations. A total of 10,650 patients comprised the modeling dataset; 7,747 were assigned to the training set and 2,903 to a held-out test set using a temporal split by diagnosis year ( ≤ 2016 vs. >2016). Using routinely available variables (age, sex, race group, Summary Stage, income group, rural–urban residence, and diagnosis period), a conventional multivariable Cox model achieved a Harrell C-index of 0.833 in the test set, indicating strong discriminative ability from standard registry variables.

**Figure 5:**
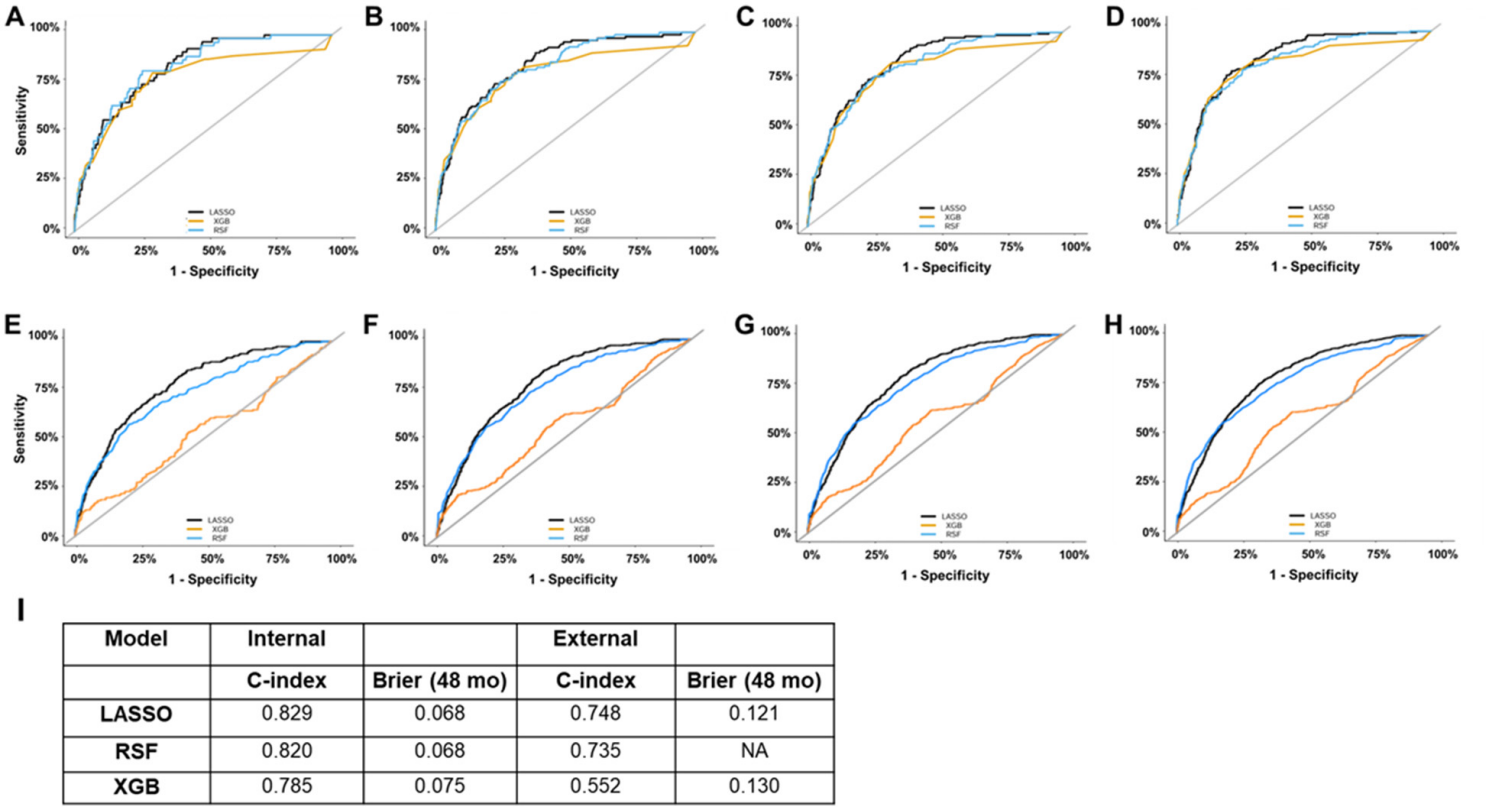
Internal and external performance of survival prediction models for mycosis fungoides. **(A–D)** Time-dependent ROC curves in the internal SEER-22 held-out test cohort at 12, 24, 36, and 48 months, respectively. **(E–H)** Time-dependent ROC curves in the external SEER-8 validation cohort at 12, 24, 36, and 48 months, respectively. Curves are shown for LASSO-penalized Cox (LASSO, black), XGBoost-Cox (XGB, orange), and random survival forest (RSF, blue); the gray 45° line indicates no discrimination. In internal validation, all three models show good discrimination across time horizons, whereas in external validation, LASSO and RSF retain discrimination while XGB approaches the diagonal, indicating limited transportability. (**I**) Summary of global discrimination and prediction error, reporting Harrell's C-index and Brier score at 48 months for internal and external validation cohorts.

All three machine-learning models, respectively LASSO-penalized Cox, RSF, and XGBoost-Cox, showed high and stable time-dependent discrimination by Uno's AUC at 12, 24, 36, and 48 months in the test set (Figure 5A–D). Uno's AUCs for LASSO-Cox were 83.2%, 83.5%, 83.6%, and 85.6% at 12, 24, 36, and 48 months, respectively; for RSF, 83.4%, 82.3%, 81.9%, and 83.7%; and for XGBoost-Cox, 77.8%, 78.6%, 79.6%, and 82.0%. Harrell C-indices were consistent with these findings (0.829 for LASSO-Cox, 0.820 for RSF, and 0.785 for XGBoost-Cox in the test cohort). Prediction error curves based on Brier scores showed lower error than the null model across follow-up for all three approaches (Figure 5I).

In 1,000 patient-level bootstrap resamples of the temporally held-out test cohort, Harrell's C-index was 0.836 (95% CI, 0.808–0.862) for LASSO-Cox, 0.832 (95% CI, 0.805–0.858) for RSF, and 0.759 (95% CI, 0.716–0.798) for XGBoost-Cox. LASSO-Cox and RSF therefore demonstrated comparable discrimination, whereas XGBoost-Cox showed lower performance.

Interpretability analyses were consistent across model families. In the RSF, age was by far the most important predictor, followed by race group, Summary Stage, rural–urban category, sex, and diagnostic period. The LASSO-Cox model retained a compact set of non-zero coefficients that mirrored these patterns: higher age, regional and distant Summary Stage, and several non-metropolitan or unknown rural codes were associated with increased hazard, whereas localized disease, more recent diagnostic period (2011+), and female sex (vs male) were associated with lower hazard (Supplementary Table 5). Overall, these findings suggest that routinely collected registry variables can support clinically interpretable risk stratification in MF, with LASSO-Cox achieving performance comparable to more complex tree-based methods.

**Web calculator**. To facilitate individualized risk estimation, we implemented the final LASSO–Cox model as a web-based calculator that returns predicted OS probabilities at 12, 24, 36, 48, and 60 months using the same covariates (age, sex, race group, Summary Stage, income group, rural–urban code, and diagnosis period). The calculator is available at: https://tzhang902.shinyapps.io/mf_calculator/

### 3.8 External validation and model transportability

We externally validated the prognostic models in an independent SEER-8 cohort defined using the same MF case definition (ICD-9700/3). After harmonizing outcomes and covariates to match the SEER-22 modeling framework, 4,753 patients were included in the external validation set. Short- to intermediate-term survival remained relatively favorable, with 12- and 36-month OS of 95.9% and 88.4%, respectively. All external models were fit using exactly the same compact predictor set as in model development (age, sex, race group, Summary Stage, income group, rural–urban code, and diagnostic period) after recoding income into shared 6-level categories and collapsing factor levels not observed in SEER-22 to “Missing.”

The LASSO-Cox model retained good time-dependent discrimination across 12, 24, 36, and 48 months (Uno's AUCs: 77.6%, 77.6%, 77.5%, and 76.4% at 12, 24, 36, and 48 months, respectively; Figure 5E–H). By contrast, XGBoost-Cox showed limited discrimination in the external cohort (Uno's AUCs 53.1%−56.7% across time points), only marginally above chance. Brier score curves were correspondingly more favorable for LASSO-Cox than for the null model over follow-up, whereas XGBoost-Cox demonstrated little to no improvement over the null model (Figure 5I).

Global discrimination metrics mirrored these results. In SEER-8, Harrell's C-index was 0.748 for LASSO-Cox and 0.735 for RSF, indicating modest attenuation from internal performance but overall preserved ranking of patient risk across registries. In contrast, the XGBoost-Cox C-index decreased to 0.552, consistent with weaker time-dependent AUC and prediction error profiles (Figure 5I). Time-specific ROC curves likewise showed stable separation for LASSO-Cox across time horizons, whereas XGBoost-Cox curves approximated the diagonal, indicating poor generalizability outside the development dataset.

## 4 Discussion

This national analysis provides a comprehensive view of how race, SES, and clinical factors collectively shape outcomes in MF. Consistent with prior reports, Black patients in our cohort exhibited higher mortality compared with White patients, even after adjusting for age, sex, stage, and SES (21). Our study adds two important observations. First, we are among the earliest to demonstrate that AI/AN patients experience similarly poor survival, with 5-year OS of 82.1%, underscoring the need for greater recognition of this underrepresented group. Second, we found that API patients exhibited the most favorable outcomes across all analyses, including 5-year OS of 92.9% and a persistently protective hazard ratio after multivariable adjustment.

The decade following 2010 marked a transition in MF management, with the introduction of histone deacetylase inhibitors and later targeted biologics such as brentuximab vedotin and mogamulizumab (22). Our temporal analyses show that survival improved modestly during this period, with statistically significant improvement among White patients, while changes within Black, API, and AI/AN groups were not statistically significant. Although population-based data cannot attribute these differences to specific treatments or policies, these findings align with broader concerns that advances in care and changes in healthcare delivery may not be equitably realized across populations. Prior work in dermatology has highlighted persistent challenges in trial representation and treatment access. For example, reports have raised concerns regarding minority enrollment in dermatology clinical trials, including CTCL studies (23), and a single-center study by Martini et al. in an ethnically diverse setting found longer delays to initiation of systemic therapy among minority patients, particularly Black individuals (24).

While individual-level biological and clinical features contribute, broader systemic inequities remain powerful drivers of disparity. Prior studies suggest that limited healthcare access, restricted medication availability, health literacy barriers, and provider or institutional biases toward minority groups play critical roles (25, 26). While prior studies have focused on survival differences, little is known about how racial disparities influence inpatient care utilization of MF. One unique aspect of our analysis was the integration of SEER and NIS datasets, a creative approach that allowed validation of survival patterns across complementary sources and exploration of inpatient care disparities. In the NIS cohort, Black patients had a longer mean hospital stay than White patients (8.9 vs. 7.2 days, *p* = 0.025; data not shown), suggesting more severe disease at presentation, delayed access to outpatient management, or systemic barriers to efficient discharge planning. Moreover, lower income and Medicaid/self-pay status were overrepresented among Black and AI/AN patients, reinforcing the link between economic disadvantage and outcome. These findings highlight how the cumulative weight of structural barriers that shapes differential survival trajectories.

The remaining disparity after adjusting for SES and stage suggests that factors intrinsic to clinical presentation and recognition play a similarly critical role (Table 2C) (27–29). For instance, MF often manifests with erythema or subtle pigmentary changes that may be more difficult to appreciate against darker skin tones. Erythematous lesions can be underrecognized in Black patients, leading to diagnostic delays, whereas hypopigmented variants (more prevalent and easily visible in patients with darker skin) are paradoxically associated with better survival outcomes in Black but not White patients (6, 30). These pigmentary differences underscore how diagnostic visibility and lesion phenotype can influence both the timing of diagnosis and the apparent prognosis.

Future research is needed to integrate molecular and immunologic profiling to explore biologic underpinnings of disparity (31–33). For example, compared with other races, Black patients of atopic dermatitis have been reported to exhibit attenuated Th1/Th17 responses and a stronger Th2 bias, with relative preservation of epidermal barrier genes such as FLG, a pattern that overlaps with MF/SS immune dysregulation (34). Moreover, small cohort studies have begun to investigate genomic alterations. Using a limited number of MF samples, Fléchon et al. (35) found that high-risk disease stages are associated with del10p11.22, gain10p15.1, gain7q, or mutations in JUNB and TET2, while shorter survival was associated with gain7q, gain10p15.1, del10p11.22, and del6q16.3. These findings raise the possibility that immunogenetic variation interacts with environmental and social factors to influence disease trajectory and therapeutic response. Limitations of this study include the lack of treatment-specific data, comorbidity information, and individual-level socioeconomic variables within SEER. Race and stage misclassification and small subgroup sizes may have constrained precision for AI/AN and API groups. Nonetheless, the consistent patterns observed across SEER and NIS strengthen the reliability of our conclusions. Future work will expand upon these findings by incorporating molecular and genomic data to delineate whether racial differences in immune regulation or tumor biology contribute to survival outcomes.

In parallel with traditional regression, we deliberately built and evaluated machine-learning survival models to determine whether more complex approaches improved prognostic performance using routinely collected registry variables. In the internal test set using SEER-22, conventional multivariable Cox regression already achieved a C-index of around 0.84, and LASSO-Cox, RSF, and XGBoost-Cox performed similarly (C ≈0.82–0.83; Uno AUC ≈0.83–0.86 through 48 months) with lower Brier prediction error than a null Kaplan–Meier–only model. This suggests that a relatively small set of core variables (age, sex, race, stage, SES, rurality, and period) already encode most of the prognostic signal available in SEER. RSF and XGBoost-Cox added non-linear and interaction structure, but offered only modest incremental discrimination in internal testing, emphasizing that more complex learners cannot compensate for the intrinsic limitations of registry data (e.g., coarse stage categories, lack of detailed treatment and comorbidity information). At the same time, the variable-importance patterns from RSF and the shrinkage pattern in LASSO converged on the same message as our fully adjusted Cox model: survival gradients are driven by age, stage, race, and socioeconomic context, with age and stage acting as broad risk “scaffolds” on which more subtle SES and geographic effects are layered. In this sense, the machine-learning analyses served less to discover completely new predictors than to corroborate and refine the structure of risk suggested by traditional modeling. Of note, adding diagnosis period did not significantly improve model fit (likelihood-ratio χ^2^ = 2.49, p = 0.115), and diagnosis in 2011–2021 was not independently associated with mortality after adjustment (HR 0.93, 95% CI 0.84–1.02). These findings suggest that diagnosis period adds little to individualized prediction beyond other covariates. Overall, machine-learning models provided limited benefit over conventional regression, and future gains in MF prognostication will likely depend on incorporating treatment, comorbidity, clinical, molecular, and socioeconomic data rather than increasing algorithmic complexity.

External validation in a non-overlapping SEER-8 registry cohort provided a stringent assessment of transportability. LASSO-Cox and RSF preserved discrimination and prediction accuracy in SEER-8, whereas the gradient-boosted model generalized poorly, consistent with known sensitivity of more flexible learners to shifts in covariate distributions and coding across datasets. Taken together, these results indicate that conventional Cox regression or a parsimonious penalized Cox model is sufficient for risk prediction using the limited set of routinely collected registry variables available in this study. LASSO-Cox was selected for implementation in the web-based calculator because it provides a compact and transparent prediction function, performs comparably to the more complex approaches, and retains performance in registry-based external validation, rather than because it substantially outperformed conventional Cox regression. The attenuation in performance from development to validation reinforces that registry-based estimates should be viewed as decision-support benchmarks rather than definitive individualized forecasts, and that further validation in clinical cohorts with richer treatment and biologic data is needed.

Several limitations should be considered. SEER does not provide sufficiently detailed treatment, comorbidity, insurance-continuity, or healthcare-access information to fully account for factors that may influence survival. Combined Summary Stage provides a broad classification of disease extent and does not reproduce the detailed TNMB staging used clinically for mycosis fungoides. Blank or unrecorded stage values were retained as an unknown/unavailable category to preserve eligible patients, but this category does not represent a distinct clinical stage and may capture differences in data availability or completeness.

In addition, socioeconomic status was approximated using county-level median household income. This ecological measure characterizes the socioeconomic context of a patient's area of residence but does not represent the income or resources of the individual patient. It may obscure substantial socioeconomic heterogeneity within counties and does not capture education, employment, household wealth, insurance continuity, transportation barriers, access to specialty care, or delays in diagnosis and treatment. Additionally, patients residing within the same county may share unmeasured contextual characteristics; because our models did not explicitly account for within-county clustering, the precision of some estimates may be affected. Race and stage may also be subject to misclassification, and the small number of AI/AN patients limited the precision of subgroup estimates. Rural–urban residence provides complementary information regarding geographic context and potential healthcare access but is not interchangeable with socioeconomic status. Consequently, residual socioeconomic and healthcare-access confounding may remain despite adjustment for county-level income.

Missing covariate data may have introduced selection bias, particularly in the prognostic modeling cohort, which required complete predictor information. Because missingness may differ across racial and socioeconomic groups, some populations may be underrepresented. We did not perform multiple imputation, and the prediction models should therefore be interpreted as applying to patients with available registry variables. Also, the proportional-hazards assumption was assessed using scaled Schoenfeld residuals and residual plots. No material violations affecting interpretation of the principal race estimates were identified. Hazard ratios were therefore interpreted under the assumption of approximately constant relative hazards over follow-up.

In summary, using national registry data, we demonstrate persistent racial/ethnic and socioeconomic inequities in MF survival in the contemporary era, with limited evidence of improvement among minority groups in the post-2010 period. These findings highlight the need for equity-focused strategies spanning early recognition, access to specialty care, and timely treatment. An externally validated, interpretable prognostic model based on routinely available variables may facilitate scalable risk stratification and support future efforts to monitor and reduce disparities in MF outcomes.

## Data Availability

The raw data supporting the conclusions of this article will be made available by the authors, without undue reservation.
