# Supplementary material for: Persistent racial and socioeconomic inequities in mycosis fungoides survival: a population-based study
Source: Front Public Health. 2026 Jul 15;14:1811186. doi: 10.3389/fpubh.2026.1811186 (PMC13416094; doi:10.3389/fpubh.2026.1811186)
Supplement: Supplementary file 1 [file Supplementary_file_1.docx]

**Supplementary Table 1. SEER database sources, registry composition, and variable definitions**

**Panel A. Database sources and study cohorts**

| **Cohort** | **SEER*Stat database** | **Diagnosis years** | **Registry composition** |
| --- | --- | --- | --- |
| Primary analytical cohort | Incidence – SEER Research Limited-Field Data, 22 Registries, November 2023 submission | 2000–2021 | All SEER-22 registriesᵃ |
| Model-development cohort | SEER-22 after exclusion of SEER-8 registries | 2000–2021 | Fourteen non-SEER-8 registriesᵇ |
| External-validation cohort | Incidence – SEER Research Data, 8 Registries, November 2024 submission | [insert exact years used] | Eight SEER registriesᶜ |

**Panel B. SEER variables and coding used in the analyses**

| **Analytical variable** | **Exact SEER*Stat variable name** | **Coding used** |
| --- | --- | --- |
| MF case definition | Site and Morphology: **ICD-O-3 Hist/behav** | 9700/3: mycosis fungoides, malignant behavior |
| Diagnosis year | **Year of diagnosis** | 2000–2021; categorized as 2000–2010 and 2011–2021 where indicated |
| Reporting source | **Type of Reporting Source** | Death-certificate-only and autopsy-only cases excluded |
| Primary sequence | **Sequence number central** | Included: one primary only or first of two or more primaries; excluded: second or later primary |
| Age | **Age recode with single ages and 90+** | Continuous age in years |
| Sex | **Sex** | Male; female |
| Race | **Race recode (White, Black, Other)** | White, Black, Asian/Pacific Islander, American Indian/Alaska Native, and Unknown |
| Summary Stage | **Combined Summary Stage (2004+)** | Localized, regional, distant, and unknown; harmonization rules described in Methods |
| Area-level income | **Median household income inflation adjusted to 2021** | Categories retained as supplied by SEER and harmonized across cohorts |
| Rural–urban residence | **Rural-Urban Continuum Code** | Categories retained or collapsed according to the prespecified modeling scheme |
| Survival time | **Survival months** | Time from diagnosis to death or last follow-up |
| Vital status | **Vital status recode (study cutoff used)** | Alive = 0; dead = 1 |

ᵃ **SEER-22 registries:** San Francisco–Oakland, Connecticut, Hawaii, Iowa, New Mexico, Seattle–Puget Sound, Utah, Metropolitan Atlanta, San Jose–Monterey, Los Angeles, Alaska Natives, Rural Georgia, California excluding San Francisco–Oakland/San Jose–Monterey/Los Angeles, Kentucky, Louisiana, New Jersey, Greater Georgia, Idaho, New York, Massachusetts, Illinois, and Texas.

ᵇ **Model-development registries:** San Jose–Monterey, Los Angeles, Alaska Natives, Rural Georgia, California excluding San Francisco–Oakland/San Jose–Monterey/Los Angeles, Kentucky, Louisiana, New Jersey, Greater Georgia, Idaho, New York, Massachusetts, Illinois, and Texas.

ᶜ **SEER-8 registries:** San Francisco–Oakland, Connecticut, Hawaii, Iowa, New Mexico, Seattle–Puget Sound, Utah, and Metropolitan Atlanta.

**Abbreviations:** ICD-O-3, International Classification of Diseases for Oncology, Third Edition; MF, mycosis fungoides; SEER, Surveillance, Epidemiology, and End Results.

**Supplemental Table 2. Comparison of Overall Survival ≤2010 vs >2010 Across Racial Groups in Mycosis Fungoides.**

|  | **race** | **time_**  **months** | **OS_before 2010** | **OS_after 2010** | **P value** |
| --- | --- | --- | --- | --- | --- |
| **1** | **American Indian/Alaska Native** | **12** | **94.7%** | **94.4%** | **0.953549740** |
| **2** | **American Indian/Alaska Native** | **36** | **84.2%** | **91.2%** | **0.468835119** |
| **3** | **American Indian/Alaska Native** | **60** | **73.7%** | **87.7%** | **0.228603879** |
| **4** | **Asian/Pacific Islander** | **12** | **96.5%** | **99.0%** | **0.023261622** |
| **5** | **Asian/Pacific Islander** | **36** | **94.2%** | **95.8%** | **0.319753005** |
| **6** | **Asian/Pacific Islander** | **60** | **90.9%** | **94.4%** | **0.084084230** |
| **7** | **Black** | **12** | **94.9%** | **95.8%** | **0.332460966** |
| **8** | **Black** | **36** | **87.6%** | **90.0%** | **0.082587186** |
| **9** | **Black** | **60** | **81.4%** | **83.6%** | **0.190089990** |
| **10** | **Unknown** | **12** | **98.9%** | **99.5%** | **0.446256350** |
| **11** | **Unknown** | **36** | **96.6%** | **98.6%** | **0.185305443** |
| **12** | **Unknown** | **60** | **94.9%** | **97.1%** | **0.256723177** |
| **13** | **White** | **12** | **96.5%** | **97.1%** | **0.110129443** |
| **14** | **White** | **36** | **89.7%** | **91.2%** | **0.016962511** |
| **15** | **White** | **60** | **84.1%** | **86.5%** | **0.002216508** |
| **16** | **Overall** | **12** | **96.3%** | **97.1%** | **1.295214e-02** |
| **17** | **Overall** | **36** | **89.8%** | **91.7%** | **4.082452e-04** |
| **18** | **Overall** | **60** | **84.4%** | **87.1%** | **4.717217e-05** |

**Supplemental Table 3. Adjusted race-by-diagnosis-era interaction analysis**

| **Result** | **HR (95% CI)** | **p-value** |
| --- | --- | --- |
| Black vs White, 2000–2010 | 1.77 (1.57–1.99) | <0.001 |
| Black vs White, 2011–2021 | 1.69 (1.46–1.96) | <0.001 |
| Black × diagnosis-era interaction | 0.96 (0.79–1.15) | 0.643 |
| Global race × diagnosis-era interaction | - | 0.1847 |

Note: Models were adjusted for age, sex, stage, and county-level income. Patients with Unknown race were excluded. The global interaction p-value was obtained using a likelihood-ratio test comparing models with and without race-by-diagnosis-era interaction terms.

## Supplemental Table 4. Income Distribution by Race

| Race Group | <$50k | $50–64k | $65–79k | $80–94k | ≥$95k | Unknown |
| --- | --- | --- | --- | --- | --- | --- |
| White | 4.8% | 16.1% | 31.3% | 19.7% | 28.0% | 0.0% |
| Black | 11.2% | 19.6% | 35.4% | 16.2% | 17.6% | NA |
| Asian/Pacific Islander | 1.1% | 5.4% | 26.0% | 22.2% | 45.4% | NA |
| American Indian/Alaska Native | 7.1% | 21.4% | 35.7% | 21.4% | 14.3% | NA |
| Unknown | 4.7% | 9.5% | 32.3% | 20.7% | 32.4% | 0.4% |

Note: Income distribution is shown as the percentage of patients within each race group. Values are based on county-level median household income quartiles linked to SEER data. NA = not available.

## Supplemental Table 5. LASSO coefficient table

| **1** | RuralCodeUnknown/missing/no match (Alaska or Hawaii - Entire State) | 1.744 | 5.72 | Higher predicted hazard |
| --- | --- | --- | --- | --- |
| **3** | SummaryStageRegional | 0.824 | 2.28 | Higher predicted hazard |
| **4** | race_ Black | 0.5637 | 1.757 | Higher predicted hazard |
| **5** | race_ American Indian/Alaska Native | 0.3454 | 1.413 | Higher predicted hazard |
| **6** | IncomeGroup$50,000 - $54,999 | 0.3268 | 1.386 | Higher predicted hazard |
| **8** | SummaryStageDistant | 0.2576 | 1.294 | Higher predicted hazard |
| **9** | IncomeGroup$45,000 - $49,999 | 0.2405 | 1.272 | Higher predicted hazard |
| **13** | IncomeGroup$60,000 - $64,999 | 0.1769 | 1.193 | Higher predicted hazard |
| **15** | IncomeGroup$55,000 - $59,999 | 0.1527 | 1.165 | Higher predicted hazard |
| **17** | RuralCodeNonmetropolitan counties adjacent to a metropolitan area | 0.0893 | 1.093 | Higher predicted hazard |
| **18** | IncomeGroup$75,000 - $79,999 | 0.0851 | 1.089 | Higher predicted hazard |
| **19** | RuralCodeCounties in metropolitan areas of 250,000 to 1 million pop | 0.0723 | 1.075 | Higher predicted hazard |
| **20** | Age | 0.0681 | 1.07 | Higher predicted hazard |
| **24** | RuralCodeNonmetropolitan counties not adjacent to a metropolitan area | 0.0464 | 1.048 | Higher predicted hazard |
| **25** | IncomeGroup$95,000 - $99,999 | 0.0412 | 1.042 | Higher predicted hazard |
| **26** | RuralCodeCounties in metropolitan areas of lt 250 thousand pop | 0.0354 | 1.036 | Higher predicted hazard |
| **29** | IncomeGroup$80,000 - $84,999 | 0.0056 | 1.006 | Higher predicted hazard |
| **30** | IncomeGroup$70,000 - $74,999 | -0.0002 | 1 | Lower predicted hazard |
| **28** | IncomeGroup$40,000 - $44,999 | -0.006 | 0.994 | Lower predicted hazard |
| **27** | IncomeGroup$90,000 - $94,999 | -0.028 | 0.972 | Lower predicted hazard |
| **23** | IncomeGroup$85,000 - $89,999 | -0.0492 | 0.952 | Lower predicted hazard |
| **22** | period2011+ | -0.0503 | 0.951 | Lower predicted hazard |
| **21** | IncomeGroup$100,000 - $109,999 | -0.0583 | 0.943 | Lower predicted hazard |
| **16** | IncomeGroup$120,000+ | -0.106 | 0.899 | Lower predicted hazard |
| **14** | IncomeGroup$110,000 - $119,999 | -0.1669 | 0.846 | Lower predicted hazard |
| **12** | SummaryStageUnknown/unstaged | -0.2074 | 0.813 | Lower predicted hazard |
| **11** | race_ Asian/Pacific Islander | -0.2195 | 0.803 | Lower predicted hazard |
| **10** | Female | -0.2398 | 0.787 | Lower predicted hazard |
| **7** | SummaryStageLocalized | -0.3238 | 0.723 | Lower predicted hazard |
| **2** | race_ Unknown | -1.0834 | 0.338 | Lower predicted hazard |


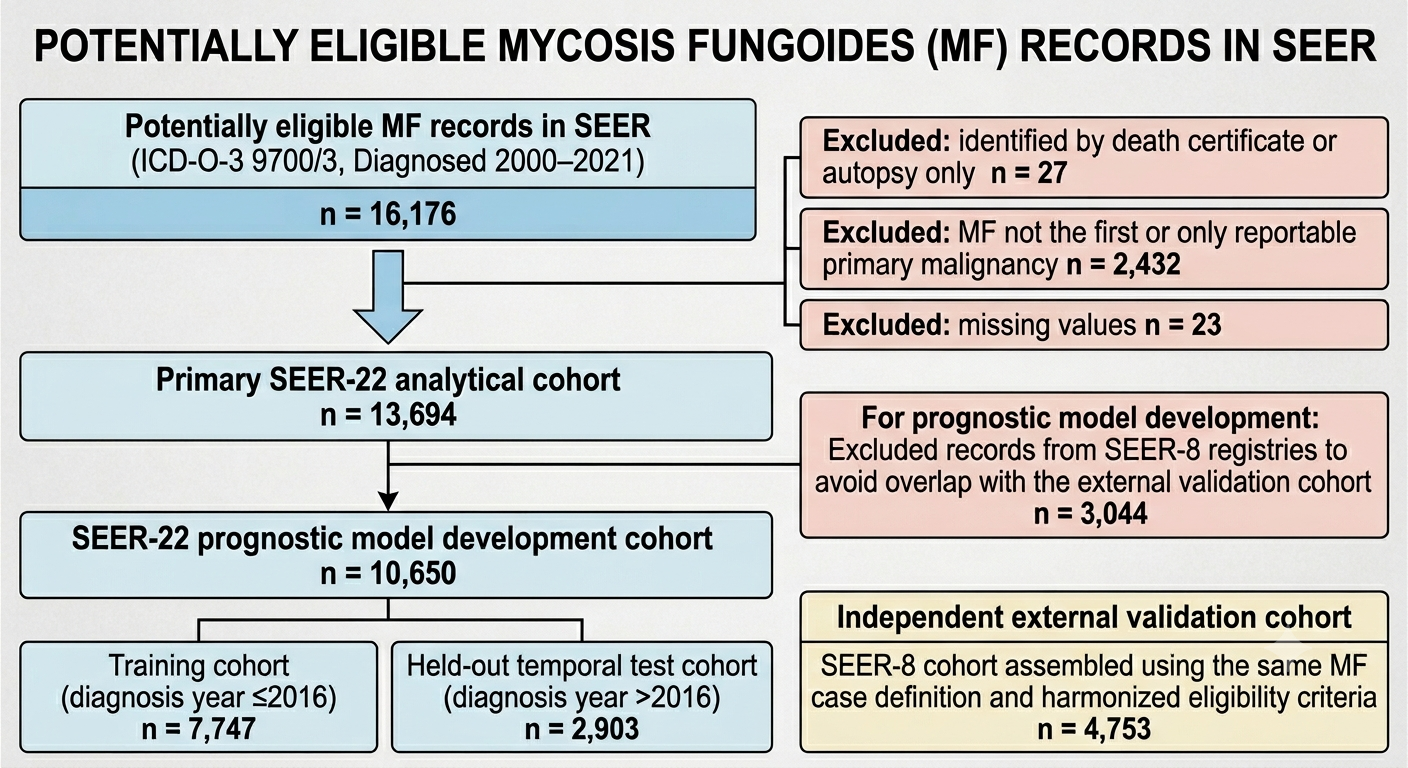


**Supplemental Figure 1. Cohort selection and allocation for disparities analyses, prognostic model development, and external validation.** Potentially eligible mycosis fungoides (MF) records were identified in SEER using ICD-O-3 histology/behavior code 9700/3 for diagnoses made from 2000 through 2021 (n = 16,176). Records identified only by death certificate or autopsy (n = 27), records in which MF was not the first or only reportable primary malignancy (n = 2,432), and records with missing required values (n = 23) were excluded, resulting in a primary SEER-22 analytical cohort of 13,694 patients. For prognostic model development, 3,044 patients from SEER-8 registries were excluded to prevent overlap with the external-validation cohort, leaving 10,650 patients. This cohort was temporally divided into a training cohort diagnosed in 2016 or earlier (n = 7,747) and a held-out test cohort diagnosed after 2016 (n = 2,903). Geographic/registry-based external validation was performed in an independent SEER-8 cohort assembled using the same MF case definition and harmonized eligibility criteria (n = 4,753). MF, mycosis fungoides; SEER, Surveillance, Epidemiology, and End Results.
