# Supplementary figures and images for: Persistent racial and socioeconomic inequities in mycosis fungoides survival: a population-based study
Source: Front Public Health. 2026 Jul 15;14:1811186. doi: 10.3389/fpubh.2026.1811186 (PMC13416094; doi:10.3389/fpubh.2026.1811186)

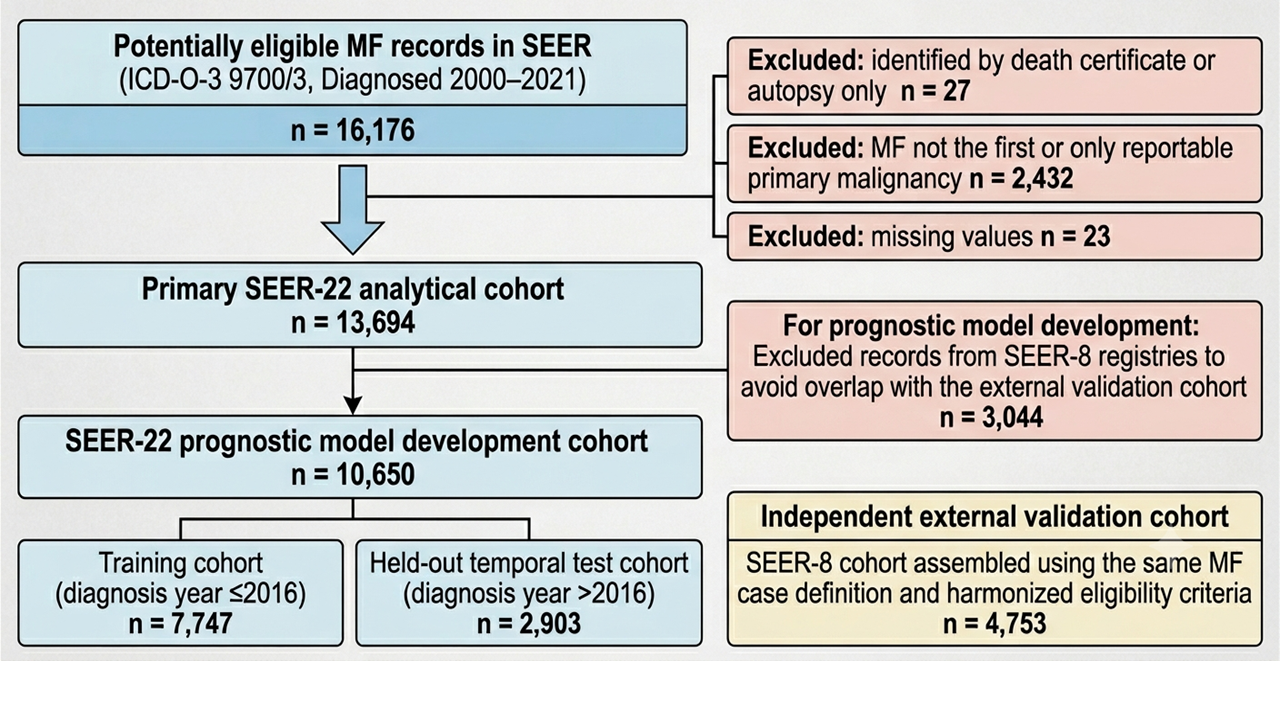

Supplement: Supplementary file 2 [file Image_1.tif]
